# Predictive Factors of Cyberbullying Perpetration amongst Spanish Adolescents

**DOI:** 10.3390/ijerph17113967

**Published:** 2020-06-03

**Authors:** Carolina Yudes, Lourdes Rey, Natalio Extremera

**Affiliations:** 1 Department of Developmental Psychology and Education, Faculty of Psychology, University of Malaga, Campus de Teatinos s/n, 29071 Málaga, Spain; 2 Department of Personality, Evaluation and Psychological Treatment, Faculty of Psychology, University of Malaga, Campus de Teatinos s/n, 29071 Malaga, Spain; lrey@uma.es; 3 Department of Social Psychology, Faculty of Psychology, University of Málaga, Campus de Teatinos s/n, 29071 Malaga, Spain; nextremera@uma.es

**Keywords:** cyberbullying, adolescence, risk factors, problematic Internet use, protective factors, emotional regulation

## Abstract

(1) Cyberbullying has gained increased attention from society and researchers due both to its negative psychosocial consequences and the problems that have risen relating to the misuse of technology. Despite the growing number of scientific studies, most research has focused on victims of cyberbullying rather than on the cyberbullies. This study examines the predictive value of personal resources (emotional intelligence, gratitude, and core self-evaluations) and risk factors (cybervictimization, problematic Internet use), and parental control in online activities on adolescents’ involvement in cyberbullying perpetration. (2) A total of 2039 Spanish adolescents between 12 and 18 years of age took part in this research (53.9% females). (3) Twenty-two percent of the sample was engaged in cyberbullying behaviors (more male adolescents). Insults and online social exclusion were the most frequent types of cyberbullying perpetration. Age, cybervictimization, problematic Internet use, and deficits in the use and regulation of emotions were the best predictors of cyberbullying perpetration. (4) Cyberbullying is a social reality in which personal and family variables converge on a particularly vulnerable age group. Our findings suggest that both well-known predictors of cyberbullying (cybervictimization and problematic Internet use) along with others less studied dimensions (i.e., emotional abilities) need to be taken into account in future school-based interventions aimed to prevent cyberbullying perpetration.

## 1. Introduction

Cyberbullying amongst adolescents is one of the many alarming problems in our current society [1,2,3]. Cyberbullying is defined as hostile and intentional behaviors of interpersonal violence repeated over time and using communication technologies (chat or instant messaging, websites, online games, etc.) [4,5,6,7,8], against peers who are unable to defend themselves [9,10]. The cyberbullies seek to emotionally harm their victims through threats, insults, and malicious teasing, spreading rumors or inciting the social exclusion, amongst other different approaches. The lack of physical and temporal limits online, in addition to easy access to contact with others and the wide and quick spread of hurtful materials [11], increase the loss of control of the victim [12], and, consequently, the negative effects on their physical and mental health. In this regard, Garaigordobil and Martínez-Valderrey [13] point out that just one or two episodes of cyberbullying are enough to produce harmful outcomes, because these incidents remain accessible to others on the Internet.

### 1.1. Cyberbullying, Problematic Internet Use and Parental Control

Despite the increase in cyberbullying research in recent years, relatively little attention has been given to the study of the psychological profile of cyberbullies. Notwithstanding, the literature has highlighted several factors as predictors of cyberbullying perpetration (for example: prior offline bullying, behavioral problems, antisocial personality, low empathy, or moral values [14]). In particular, three of the most studied factors reveal a strong positive relation with cyberbullying perpetration: the experience of being victim of cyberbullying [2,3,15,16], the parental control of adolescents’ online activities [17,18], and a problematic Internet use [19,20].

Focusing on these possible risk factors, cyberbullying victimization is a key predictor of cyberbullying perpetration [16,21]. From the point of view of the victim, one reason to bully back in return is the variety of negative emotions they feel, for example, shame, anger, or frustration. Those emotions might lead adolescents to feel powerful and take revenge [22].

Regarding parental control, individuals who are exposed to media content without supervision (for example: setting rules, guide on online risks, privacy, inappropriate Internet use) may be affected by their attitudes towards cyberbullying [23]. Low parental supervision is a significant risk factor for cyberbullying, especially if adolescents also feel such parental control as restrictive or intrusive [17,19]. These observations are empirically supported by Chang et al. [24], who found that adolescents who perceive their parents’ supervision as light are more likely to be involved in cyberbullying, as well as in other risk-related behaviors, for example, problematic Internet use and substance use.

Finally, concerning problematic Internet use, adolescents who are heavy Internet users might behave in an uncontrolled way, for example, by acting aggressively in their online interpersonal relationships, or reacting more violently to provocations and comments, which may lead to an increased risk of cyberbullying [20]. The potential explanations for this relation include the addictive properties of the Internet when it is used as a standard strategy to reduce frustration, anger, or anxiety [25], the effect of exposure due to both the number and nature of the websites visited [26] or the online disinhibition effect when compared to offline behavior [27]. 

Traditionally, models that attempt to explain cyberbullying perpetration have included these mentioned factors in addition to other demographic and contextual variables. Nonetheless, meta-analyses carried out by Chen et al. [28] highlight that other individual or interpersonal factors could be also significant but have been less studied to date. Most importantly, one of the shortcomings observed throughout the studies on cyberbullying perpetration is the focus on negative individual variables (or risk factors). Accordingly, the study of the predictors of cyberbullying remain incomplete. Further research is needed to incorporate new potential predictors (cognitive, emotional, and contextual), and develop more comprehensive models to explain the cause of cyberbullying.

### 1.2. Cyberbullying and Personal Positive Resources

According to theoretical and empirical considerations, this research contemplates that certain positive personal resources, such as emotional intelligence (EI), gratitude or core self-evaluation (CSE), may be candidate factors in reducing the likelihood of cyberbullying perpetration [22,29,30,31].

The scientific literature has conceptualized EI as the capacity to process emotional information accurately and efficiently, and to perceive, understand, and regulate emotions [32,33]. Recently, studies have emphasized that the ability to process and manage emotional information has an impact on both adaptive functioning [34] and on a range of disruptive behaviors in schools [35], including cyberbullying [36,37].

Similarly, gratitude is defined as a personality-based proneness to experience grateful emotion [38]. Grateful people typically attribute positive outcomes to the actions of others, and frequently behave in a prosocial manner toward others, even when doing so is costly to the self [39]. This moral characteristic might make adolescents less inclined to find motives to become angry and aggressive in school [40].

CSE is another dispositional factor that may reduce aggressiveness. This is defined as peoples’ fundamental evaluations of their worth, competence, capabilities, and functioning in their environment. More specifically, CSE is a high-order construct reflected in four lower-order traits: global self-esteem, locus of control, neuroticism, and generalized self-efficacy [41]. In general, it is assumed that personal beliefs influence individuals’ behaviors and, therefore, might determine involvement in cyberbullying above and beyond normal patterns of Internet use [42,43]. While no research has been conducted in cyberbullying at school using CSE as a key dimension, some preliminary findings have found the potential influence of the specifics traits on behavioral problems, such as violence: self-esteem [8,44], locus of control [45], self-efficacy [46], and neuroticism [47]. These findings are consistent with the theoretical proposal that peoples’ appraisals of their fundamental self-worth and capabilities constitute personal resources that might limit the development of antisocial and aggressive behaviors.

In sum, despite there being empirical evidence of the relationship between these robust well-known predictors (that is, being a victim of cyberbullying, problematic Internet use, and parental control) in cyberbullying perpetration, further research is needed to incorporate new potential predictors and develop more comprehensive models to explain the cause of cyberbullying. Moreover, understanding how these predictors influence violent behaviors might be a step forward in addressing the issues for school counselors who work to prevent cyberbullying, and who may be interested in designing programs focused on promoting positive qualities in adolescents.

### 1.3. Present Study

This research aims to contribute to the current literature in cyberbullying perpetration and incorporates the study of three positive personal resources (EI, gratitude, and CSE). Accordingly, we examine whether these potential predictors offer explanatory power in the prediction of cyberbullying perpetration relative to other well-established factors (cybervictimization, problematic Internet use, and parental control). 

The purpose of the present study was to explore personal and family variables that could predict cyberbullying perpetration in a sample of Spanish adolescents. Specifically, the aims are three-fold: (a) to study the prevalence of cyberbullying perpetration and different types of aggression amongst female and male adolescents; (b) to examine the associations between cyberbullying perpetration and personal and family variables of interest (cybervictimization, problematic Internet use, parental control, EI, gratitude, and CSE); and (c) to analyze the effects of these variables on cyberbullying perpetration.

Consistent with previous findings [48], we expect to find a strong correlation between cyberbullying perpetration and victimization, problematic Internet use, and parental control [2,15,23]. Finally, in keeping with the notion that personal resources might help to mitigate the likelihood of an adolescent becoming a cyberbully, we expected to find evidence for the predictive value of emotional abilities, gratitude, and CSE as buffers of cyberbullying perpetration.

## 2. Materials and Methods

### 2.1. Participants

A total of 2085 adolescents (females: 1120 and males: 965) between 12 and 18 years of age were recruited from six secondary schools in Malaga (province of Andalusia, south of Spain) for this study. The education levels were distributed between the first and fourth year of compulsory secondary education and the first and second year of high school. Those participants with incomplete questionnaires were removed from the analyses, which resulted in a final sample of 2039 adolescents (mean age = 14.6; *SD* = 1.6; females: 53.9%; males: 46.1%).

### 2.2. Measures

#### 2.2.1. Dependent Variables

Cyberbullying was measured by using the European Cyberbullying Intervention Project Questionnaire (ECIPQ; [49,50]). ECIPQ is a self-reported measure that evaluates cyberbullying frequency over the two months prior. It is composed of 11 items for cyberbullying perpetration and 11 for cyberbullying victimization. Participants are required to answer for their involvement in different physical, verbal, psychological cyberbullying behaviors (for example, “I have threatened someone through text message (SMS) or Internet messages”; “Someone has said foul words about me or insulted me using email or SMS”). Items are answered on a 5-point Likert-type scale (from 0 = never to 4 = more than once a week). Cronbach’s alpha was 0.76 and 0.83 for cyberbullying perpetration and cybervictimization, respectively.

#### 2.2.2. Independent Variables

Problematic Internet use was evaluated using the Internet Addiction Test (IAT, the expanded version of the Internet Addiction Diagnostic Questionnaire, IADQ [51,52]). It examines the impact of Internet use on daily routines, social life, or feelings. This test consists of 20 items rated on a 5-point Likert-type scale (from 1 = rarely to 5 = always) with a minimum score of 20 and maximum of 100, higher scores indicate problematic Internet use [51]. Example of items included: “How often do others in your life complain to you about the amount of time you spend online?”; “How often do you snap, yell, or act annoyed if someone bothers you while you are online?”. The Cronbach’s alpha for this study was 0.87.

Parental control of adolescents’ online activities was assessed, in a similar way to other previous studies [53,54], using two questions. One of them referred to home use of the Internet (“At home, how much do your parents monitor what you are doing on the Internet?”) and the other to Internet use outside (“How much do your parents know what you are doing on the Internet when you use it outside the home?”). The answers were requested in a 5-point Likert-type scale (from 1 = never to 5 = always). Higher scores indicate more parental supervision of Internet use. 

Emotional intelligence was measured with the Wong and Law Emotional Intelligence Scale (WLEIS; [55,56]). This scale is composed of 16 self-report short statements measuring four dimensions: self-emotion appraisal (SEA, e.g., “I have a good sense of why I have certain feelings”), others-emotion appraisal (OEA, e.g., “I always know my friends’ emotions from their behavior”), use of emotion (UOE, e.g., “I would always encourage myself to try my best”) and regulation of emotion (ROE, e.g., “I am able to control my temper and handle difficulties rationally”). Each item is rated on a 7-point Likert-type scale (from 1 = totally disagree to 7 = totally agree). The items are all positively worded so that higher scores indicate higher EI. Each dimension was considered separately for this study. The internal consistency reliability coefficients of each dimension were SEA = 0.77; OEA = 0.73; UOE = 0.76 and ROE = 0.78.

Gratitude was measured with the Gratitude Questionnaire (GQ; [38,57]). The Spanish version of this scale is a 5-item self-report measure of grateful disposition in daily life. Example of items included: “I have so much in life to be thankful for”. It uses a 7-point Likert-type scale (from 1 = strongly disagree to 7 = strongly agree). The Cronbach’s alpha was 0.77.

CSE were assessed with the Core Self-Evaluations Scale (CSES; [58]), a 12-item scale in order to measure the underlying self-evaluative factor reflected in items as for example: “My life is determined by my own actions”. It uses a 5-point Likert-type scale (from 1 = strongly disagree to 5 = strongly agree). The CSE had good psychometric properties in the Spanish adolescent population [58,59]. The internal consistency of the total score was 0.77.

### 2.3. Procedure

The data for this work came from a larger project on personal protective factors, wellbeing and use of new communication technologies amongst adolescents. 

A convenience sampling method was used to contact schools. Data collection was carried out during one-hour tutorials between April and June 2018. A passive consent procedure was employed. The head teachers informed parents about the general purpose of the study, and they were asked to contact the schools if they did not want their child(ren) to participate. Participation was anonymous. 

### 2.4. Data Analyses

The data were analyzed using SPSS statistics software package, version 25 (IBM Corp., Armonk, NY, USA). The missing data were weighted (less than 1%). When differences were statistically significant, Cohen’s d was computed to estimate the effect size. 

First, descriptive analysis of the frequency of cyberbullying perpetration behaviors was performed to study differences between males and females. Following that, bivariate correlation analyses (Pearson or Spearman’s coefficients, depending on the type of variable) was calculated to study the degree of association amongst the variables of the study. Subsequently, a logistic regression analysis was conducted to determine the predictive value of each variable included in the study. Finally, a binary logistic regression analysis was used to identify which of the examined predictors were the most important variables dimensions for distinguishing between cyberbullies and non-cyberbullies group. 

### 2.5. Ethical Approval

The study was carried out in accordance with the ethical principles for psychological research involving human subjects and with guarantees of voluntariness and data anonymity. The Research Ethics Committee of the University of Malaga (62-2016-H) approved the research.

## 3. Results

### 3.1. Descriptive Analyses

#### 3.1.1. Frequency and Types of Cyberbullying Perpetration

As noted in Table 1, less than a third of participants (22%, *n* = 448) had been involved in cyberbullying perpetration behaviors in the last two months. Consistent with previous findings, male adolescents reported more participation than females. Moreover, it is important to highlight that 25.7% (*n* = 525) of the sample was involved as a victim of cyberbullying. These percentages were estimated based on the responses in the ECIPQ; the participants who reported two or more in at least one item of the questionnaire were added to the group of cyberbullies. The next step was to calculate the number of different approaches (or types of cyberbullying-related behavior) used by these cyberbullies. Almost half of these adolescents were involved in only one approach (a detailed distribution by number and gender can be seen in Table 1).

**Figure 1:**
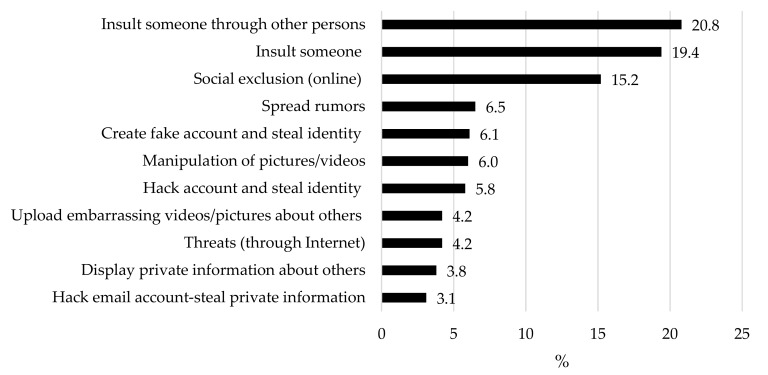
Types of cyberbullyingrelated behavior in cyberbullies sample. Notes: For this estimation, only the percentages from “2-Yes, once or twice a month” onwards were included. The same participant can be involved in different types therefore the computed is not tally to 100%.

Regarding the frequency of each approach in cyberbullies, Figure 1 shows that the most frequent type were the insults (40.2% considers both “insults about other persons said to others via the Internet or SMS messages” and “direct personal insults via email or SMS messages”) and online social exclusion (15.2%).

#### 3.1.2. Correlation between Cyberbullying Perpetration and Other Measures

Table 2 shows Pearson’s and Spearman correlations between cyberbullying perpetration and the other measured variables. These were computed for all participants. The results showed that cyberbullying perpetration was positively related to cybervictimisation and to problematic Internet use (moderately). It was significantly weakly negatively associated with all other measures (*r* range −0.07 to −0.18, *p* < 0.01).

#### 3.1.3. Predictors of Cyberbullying Perpetration

Logistic regressions were performed to identify the odds of being a cyberbully based on the independent variables considered. Age and sex were included as covariables to control spurious relationships. All the potential predictors were entered into the equation simultaneously. The variance inflation factors ranged from 1.13 to 1.97 in such a way that there was no evidence of multicollinearity. As seen in Table 3, the obtained model was a good fit because it explained 44% of the variance of cyberbullying perpetration. Eight of the 10 studied variables, including gender and age, predicted cyberbullying perpetration in the model final. The results indicated that cybervictimization, problematic Internet use as well as deficits in some EI dimensions and low parental control increased the likelihood of cyberbullying perpetration.

#### 3.1.4. Binary Regression Analyses

The next stage of analysis was to estimate a binary logistic regression model using two categories of cyberbullying perpetration as the dependent variable (0 = non-cyberbullies; 1 = cyberbullies or those participants who scored 2 or more in the ECIPQ) and all other studied variables as independent variables. The prediction success was 82.7%. The results indicated a small to moderately strong model predicting perpetration (χ^2^ = 481.53; *p* < 0.001); the Nagelkerke’s R^2^ was 33.9. The Wald criterion demonstrated that the most robust predictor was cybervictimization (odd ratio = 12.07, *p* < 0.001) but age (odd ratio = 1.22, *p* < 0.001) and problematic Internet use (odd ratio = 1.03, *p* < 0.001) were significant. Within the protective variables, only emotional regulation and use of emotion were significant (odd ratio = 0.79, *p* < 0.001; odd ratio = 1.21, *p* < 0.01, respectively) (see Table 4).

## 4. Discussion

While considerable research has aimed to evaluate the predictors of cyberbullying victimization, relatively little research has focused upon the predictors of cyberbullying perpetration. Therefore, this study aimed at identifying the predictive factors of cyberbullying perpetration in a sample of Spanish adolescents.

The aim was to contribute to the research literature by (a) examining the prevalence of cyberbullying perpetration and different types of cyberbullying-related behavior amongst female and male adolescents; (b) analyzing the associations between cyberbullying perpetration and personal and family variables of interest (cybervictimization, problematic Internet use, parental control, EI, gratitude, CSE); and (c) exploring the effects of these variables to predict cyberbullying perpetration.

In regards to prevalence, 22% of the sample was considered to consist of cyberbullies. This finding was consistent with the results of a recent systematic review of meta-analyses [48]. The most frequent cyberbullying-related behavior reported was to insult, which again was similar to previous studies [40,60,61]. Furthermore, and consistent with previous research, our results showed that males were more involved in cyberbullying perpetration than females [3,15,17,62,63]. However, nowadays, findings about the role of gender in cyberbullying are not clear, and although more recent research support an equal involvement in boys and girls as cyberbullies [64], it could be depending on age [65] or other personal experiences [66].

With respect to associations between variables of interest, cyberbullying perpetration was positively related to cybervictimization [2,3,15,16,67] and problematic Internet use [14,15,68]. It was negatively associated with parental control on online activities, CSE, gratitude, and EI. 

However, regarding the third aim, the results of the binary logistic regression showed that the most important predictors were in this order: cybervictimization, age, problematic Internet use and deficits in use and emotional regulation. 

Supporting these findings, the probability of being involved in cyberbullying perpetration is nine times greater in victims of cyberbullying than in non-victims [21]. This relationship between both roles may be result of cyberbullies’ internal motivations, such as redirecting feelings and taking revenge to counter hurt [69] or deficits in emotional regulation and expression [22]. 

The second significant predictor of cyberbullying perpetration was age. Specifically, older adolescents obtained higher scores in cyberbullying perpetration than younger (early adolescents: X = 0.15, *SD* = 0.29; late adolescents: X = 0.26, *SD* = 0.37; *p* < 0.001). The percentage of those cyberbullied aged between 15 and 18 years of age (28.1%) was double that of 12 to 14 years old (14.9%). One explanation for this is related to the other predictors found in this study, parental control, and problematic Internet use. In this sense, early adolescents have more limited and controlled access to the Internet by their parents [70]. The findings suggest that parental control or monitoring online behaviors can be both a protective factor and a vulnerability factor [19,71]. In our results, although parental control did not appear as predictive in the binary regression analysis, a negative and significantly correlation with cyberbullying perpetration was observed. On the other hand, people who were connected for more time, especially in social media [72], had the highest probability of engaging in cyberbullying behaviors (as the bully) [2,24,53,68,73]. This aggression-exposure process is cyclical, so that longer time spent online leads to more cyberaggression, and vice versa [74]. In addition, some authors have suggested that cyberbullying is an indirect aggression that develops as the person engages in it. Thus, it is more frequent (and complex) when children and adolescents mature and develop better skills in social settings [75]. An online disinhibition effect [27], according to which cyberbullies fail to take responsibility for their actions and do not even perceive them as harmful because the effects are not seen [66], can result in minimal empathy or moral disengagement with the victim. Accordingly, more specific questions concerning parental control and strategies carried out depending on the age could have evidenced more accurate explanations about this relation.

Finally, the last of the predictors was EI, in particular, low levels in use and regulation of emotions. Previous research found that adolescents with better emotional abilities show less risk behaviors, that is, aggressive [35] or antisocial behaviors [76], and other such as substance abuse or depression [77]. Furthermore, cyberbullies scored low in social and emotional competencies [37]. In general, adolescents with higher scores on the positive social behavior scale showed high EI generally and scored highly for emotion regulation in particular [78].

In conclusion, the findings in this study contribute to the literature on predictive factors of cyberbullying perpetration. Protective factors include emotional abilities (in particular, use and emotional regulation) and vulnerability factors include cybervictimization and problematic Internet use. These have hitherto only been studied in isolation, but in combination they can offer a more comprehensive explanation as to why adolescents may or may not become cyberbullies. They confirm the need to apply educational programs that support the teaching of socio-emotional competencies and the responsible use of technologies. In this digital age, preventive and consciousness-raising interventions by schools and families are necessary to reduce cyberbullying behaviors.

### Limitations and Future Research

This study has several limitations. A major one is the self-reported nature of the data. The findings may have been vulnerable to typical bias, such as common method variance systematic response distortions, and specifically social desirability bias in the answering of questions. Additionally, parental supervision was assessed using only two general questions about parental control. Taking into account the potential connection amongst parental control, problematic Internet use and involvement in cyberbullying-related behavior, in order to replicate our findings, future studies should include some well-validated measures of this variable, even including other significant dimensions such as time limits or types of Internet usage rules. Another shortcoming is the cross-sectional design, which did not permit for causal statements. To add to the validity of the findings, future studies should consider collecting multisource data using longitudinal and experimental design. A further consideration is the incidental sampling design that was used to recruit the adolescents, a choice that may have reduced the general validity of the results. Future studies using a random sampling design would strengthen the findings. More research needs to be conducted to explore the impact of other key factors in cyberbullying perpetration behaviors. Additional potential predictors, such as big five traits, dispositional aggression or cognitive intelligence should also be included to examine more integrative and comprehensive models that might serve as a framework for researchers and school practitioners to counter cyberbullying. Further studies should consider whether these personal characteristics are the most influential predictors in cyberbullying or in traditional bullying perpetration.

## 5. Conclusions

The present study is the first to examine the specific role of personal resources (emotional intelligence, gratitude, and CSE) as predictors of cyberbullying perpetration amongst Spanish adolescents, with gender, cybervictimization, parental control, and problematic Internet use as confounding variables. Although it was found that most of the personal resources were significantly associated with lower cyberbullying perpetration, specific regression analysis showed that the most important predictors were cybervictimization, problematic Internet use, and low EI. Pending replication, these preliminary findings suggest that deficits in emotional abilities account for additional unique variance in cyberbullying perpetration. Accordingly, the results underscore a need for researchers to develop more complex models of cyberbullying perpetration in adolescence that take into consideration not only prior background variables, but also the presence or absence of different personal resources. Efforts to reduce cyberbullying perpetration amongst Spanish adolescents should concentrate on modifying their Internet use habits, intervening when they have experienced prior victimization, and helping them to develop higher emotional abilities.

## Figures and Tables

**Table 1:** Frequencies of cyberbullying perpetration behaviors in the last two months.

Number of Approaches	Overall Sample (*n* = 2039)	Males (*n* = 940)	Females (*n* = 1099)
None approach	78%	75.4%	80.3%
**Number of Different Approaches Used in Cyberbullies**	**Overall Sample** **(22%; *n* = 448)**	**Males** **(24.6%; *n* = 231)**	**Females** **(19.7%; *n* = 217)**
One approach	49.6%	45.5%	54.4%
Two to four approaches	42%	44.6%	39.2%
Five to seven approaches	5.1%	6.5%	4.1%
Eight or more approaches	3.3%	4.3%	2.3%

**Table 2:** Correlation coefficients of the variables in study.

Dimensions	1	2	3	4	5	6	7	8	9	10
Cyberbullying perpetration	--									
Cybervictimization	0.66 **									
Problematic Internet use	0.29 **	0.28 **								
CSE	−0.18 *	−0.24 **	−0.37 **							
Gratitude	−0.16 **	−0.18 **	−0.20 **	0.35 **						
SEA	−0.13 **	−0.14 **	−0.15 *	0.36 **	0.35 **					
OEA	−0.07 **	−0.01	0.005	0.02	0.26 **	0.43 **				
UOE	−0.11 **	−0.14 **	−0.20 **	0.47 **	0.41 **	0.55 **	0.39 **			
ROE	−0.14 **	−0.15 **	−0.19 **	0.44 **	0.30 **	0.62 **	0.29 **	0.55 **		
Parental control (at home)	−0.21 **	−0.14 **	−0.15 **	0.12 **	0.10 **	0.05 *	0.05 *	0.14 **	0.09 **	
Parental control (outside)	−0.20 **	−0.13 **	−0.22 **	0.12 **	0.10 **	0.06 **	0.05 **	0.11 **	0.08 **	0.55 **

Notes: * *p* < 0.05; ** *p* < 0.01. CSE= core self-evaluations, SEA = self-emotion appraisal, OEA = others-emotion appraisal, UOE = use of emotion, ROE = regulation of emotion, CSE = core self-evaluation.

**Table 3:** Results of logistic regression for predicting cyberbullying perpetration.

Cyberbullying Perpetration	R^2^	F	ΔR^2^	β	SE	95% CI	
	44.5	127.9 ***	44.2				
**Predictor**						**Lower** **Limit**	**Upper** **Limit**
Sex				−0.06 **	0.012	−0.07	−0.02
Age				−0.06 **	0.004	0.01	0.02
Cybervictimization				0.59 ***	0.014	0.44	0.50
Problematic Internet use				0.12 ***	0.001	0.00	0.00
Parental control (at home)				−0.02	0.005	−0.02	0.00
Parental control (outside)				−0.04 *	0.006	−0.02	0.00
SEA				−0.002	0.006	−0.01	0.01
OEA				−0.05 *	0.006	−0.02	0.00
UOE				0.05 *	0.006	0.00	0.03
ROE				−0.05 *	0.006	−0.02	0.00
Gratitude				−0.01	0.006	−0.02	0.01
CSE				0.02	0.012	−0.01	0.04

Notes: * *p* < 0.05, ** *p* < 0.01; *** *p* < 0.001. SE: Standard error. SEA = self-emotion appraisal, OEA = others-emotion appraisal, UOE = use of emotion, ROE = regulation of emotion.

**Table 4:** Binary logistic regression analyses predicting cyberbullying perpetration.

Predictor	*β* (SE)	Wald	*p*	Odds Ratio
Sex	−0.36 (0.14)	6.59	0.010 *	0.70
Age	0.20 (0.05)	19.28	0.000 ***	1.22
Cybervictimization	2.49 (0.19)	176.87	0.000 ***	12.07
Problematic Internet use	0.04 (0.01)	37.89	0.000 ***	1.04
Parental control (at home)	−0.09 (0.07)	1.49	0.221	0.92
Parental control (outside)	−0.11 (0.06)	3.32	0.069	0.89
SEA	0.08 (0.07)	1.36	0.243	1.09
OEA	−0.10 (0.06)	2.19	0.139	0.91
UOE	0.19 (0.06)	8.49	0.004 **	1.21
ROE	−0.23 (0.06)	12.56	0.000 ***	0.79
Gratitude	0.00 (0.07)	0.00	0.992	0.99
CSE	0.10 (0.14)	0.53	0.467	1.11

Notes: * *p* < 0.05, ** *p* < 0.01; *** *p* < 0.001. SE: Standard error. SEA = self-emotion appraisal, OEA = others-emotion appraisal, UOE = use of emotion, ROE = regulation of emotion. Odds ratio is the measurement of probability (the more the odds ratio differs from 1, the stronger the association).
